# A case and literature review of hypokalemic periodic paralysis associated with heavy cola consumption

**DOI:** 10.3389/fnut.2026.1841484

**Published:** 2026-08-04

**Authors:** Wen Chen, Yi Pang, Junlin Qiu

**Affiliations:** 1 Department of Endocrinology, Beihai People’s Hospital, The Ninth Affiliated Hospital of Guangxi Medical University, Beihai, Guangxi, China; 2 Guilin Medical University, Guilin, Guangxi, China

**Keywords:** cola, hypokalemia, olanzapine, periodic paralysis, schizophrenia

## Abstract

This clinical case report involves a 34-year-old male patient with a history of type 2 diabetes and schizophrenia, who took oral olanzapine for a long time and visited the hospital several times because of lower limb fatigue caused by hypokalemia. Although the symptoms were alleviated by potassium supplementation, they recurred. The patient was hospitalized because of hypoglycemia and fatigue. Laboratory examination revealed hypokalemia, After supplementing potassium, the symptoms gradually eased. To prevent the patient from experiencing hypokalemia again, we asked about his diet and found that he had an unbalanced diet with a lot of cola consumption. To confirm that heavy cola drinking and an unbalanced diet might be related to hypokalemic periodic paralysis, we advised the patient to stop drinking cola and maintain a balanced diet. Follow-up studies showed that after stopping cola and eating a balanced diet, the patient did not experience hypokalemia again. This suggests that drinking a lot of cola is related to episodes of hypokalemic periodic paralysis. Therefore, when doctors encounter patients with hypokalemic periodic paralysis in routine practice, they should pay attention to the patient’s eating habits during consultations to avoid missing a diagnosis and recurrence.

## Introduction

Although hypokalemic periodic paralysis is relatively common, periodic paralysis caused by drinking a lot of cola is relatively rare. Since the first report of cola-induced hypokalemia in 1994 (1), multiple cases of hypokalemia caused by consuming large amounts of cola have been reported (2–12). The consumption of beverages like cola and the risk of low potassium caused by drinking large amounts of cola have increased in recent years. Here, we report a case of hypokalemic periodic paralysis caused by long-term drinking of large amounts of cola in a patient who also had type 2 diabetes mellitus. Although the patient often had hypokalemia-induced lower limb fatigue, which improved after in-hospital potassium supplementation, the cause was not examined. After the patient was admitted to the hospital with hypoglycemia because of oral hypoglycemic drugs and low food intake, the hypoglycemia was soon corrected. Muscle strength returned to normal after treatment with potassium supplements, but the patient had fatigue and limited walking, with fatigue symptoms disappearing after 2 months. Moreover, the patient could walk normally after continuous potassium supplementation and correcting the habit of eating less staple food, which was caused by drinking large amounts of cola.

## Report of a case

A 34-year-old male patient was diagnosed with type 2 diabetes mellitus 7 years ago. The patient was given metformin sustained-release capsules, glimepiride tablets, and pyrrolidone tablets to reduce the blood glucose, which fluctuated from 8 mmol/L to 13 mmol/L. Twelve hours after taking oral hypoglycemic drugs, the patient ate less but was rushed to the internal medicine emergency department, with shaking hands and irritability, for treatment. A glucose test revealed a blood glucose level of 2.1 mmol/L, which improved after sugar supplementation. It recurred after 6 h, accompanied by lower limb fatigue. The patient had a history of recurrent hypokalemia leading to lower limb weakness for 6 years (which improved after potassium supplementation), a 10-year history of schizophrenia, and had been taking oral olanzapine.

Physical examination at admission revealed the following results: Body temperature: 36.9 °C, Heart rate: 117 times/min, Breathing: 20 times/min, Blood pressure: 110/64 mmHg, Grade 5 muscle strength in both upper limbs, Grade 4 muscle strength in both lower limbs, negative pathological reflexes, and meningeal irritation signs. Emergency examination revealed the following results: pH 7.48 (blood gas analysis), blood creatinine level of 117 μmol/L, blood glucose level of 8.45 mmol/L, K^+^ level of 2.74 mmol/L, Na^+^ level of 129.2 mmol/L, Cl^–^ level of 90 mmol/L, and Creatine Kinase level of 453 U/L. Laboratory tests after admission revealed the following results: for liver function, globulin level of 15.2 g/L, albumin level of 31.8 g/L, total serum protein level of 47 g/L, triglyceride level of 1.81 mmol/L, Na^+^ level of 128.4 mmol/L, and K^+^ level of 3.01 mmol/L; Hemoglobin level of 106g/L; for thyroid function, blood cortisol, renin, and aldosterone levels were normal, while OGTT, C-peptide release test, 24-h urinary potassium, and 24-h urine microalbumin results were 7.56–12.62–17.00–14.21 (mmol/L), 0.88–1.14–1.78–2.11 (pmol/mL), 25.2 mmol/L, 30.13 and mg/24-h urine output, respectively. Electrocardiogram revealed sinus tachycardia and T-wave changes. Fundus photography revealed exudation in the right eye. Peripheral neurogenic damage was seen in the electromyogram of the lower limbs. Chest X-ray and cranial magnetic resonance imaging revealed no obvious abnormality. Color Doppler ultrasound revealed fatty liver, liver cyst, and gallstones, but no splenic abnormality. There was no abnormality in the kidneys, ureters, and bladder. There was prostate calcification. Adrenal color Doppler ultrasound revealed no abnormality.

The patient’s hypoglycemia was corrected soon after they received supplemental glucose and discontinued the hypoglycemic drugs. The patient’s medication and diet were tracked in detail since blood glucose control is poor in patients with multiple chronic complications of type 2 diabetes and hypoglycemia. Despite taking medication on time, the patient drank coca-cola (0.12 mg/mL caffeine content) for a long time (>4000 mL daily) and rarely ate staple food. The patient gradually stopped drinking cola, and their blood glucose was controlled well after the need for diet control and adjusting poor dietary habits to control blood glucose was explained to the patient and his family. The patient was given intravenous and oral potassium supplementation because of hypokalemia, which quickly restored serum potassium levels with gradual muscle strength recovery. However, an investigation of the causes of hypokalemia did not reveal any evidence of hyperthyroidism or adrenal disease. The lower limb fatigue caused by hypokalemia occurred several times and was corrected by potassium supplementation, which is consistent with the diagnosis of hypokalemic periodic paralysis.

**FIGURE 1:**
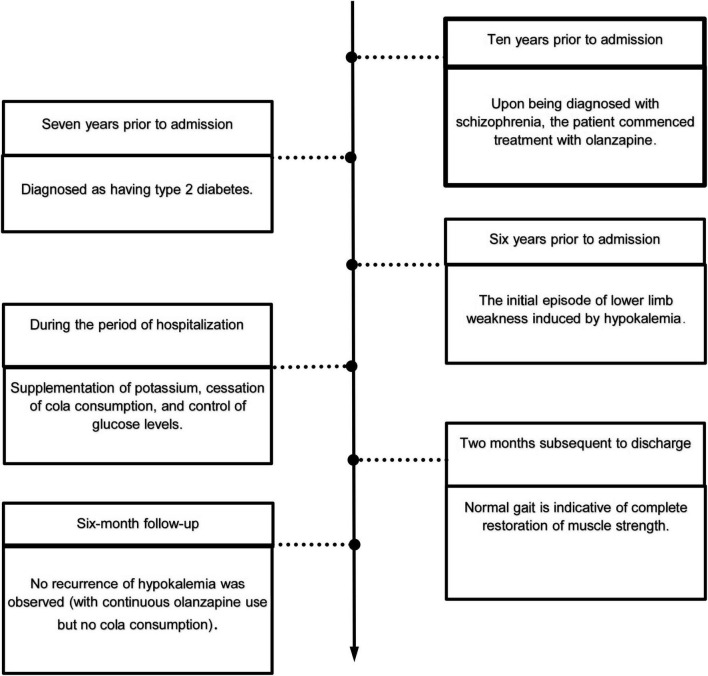
Timeline of the patient’s history and management.

The patient could walk normally during follow-up after 2 months, without any lower limb fatigue. The patient did not have lower limb fatigue during follow-up half a year later, during which they had been taking oral olanzapine but not cola, and their blood glucose was stable. During this follow-up, according to his family, he was a bit resistant to not being able to drink cola, but as his lower limb strength improved and he no longer had low potassium, he listened to his family’s advice and gave up cola (Figure 1).

## Review of the literature

**FIGURE 2:**
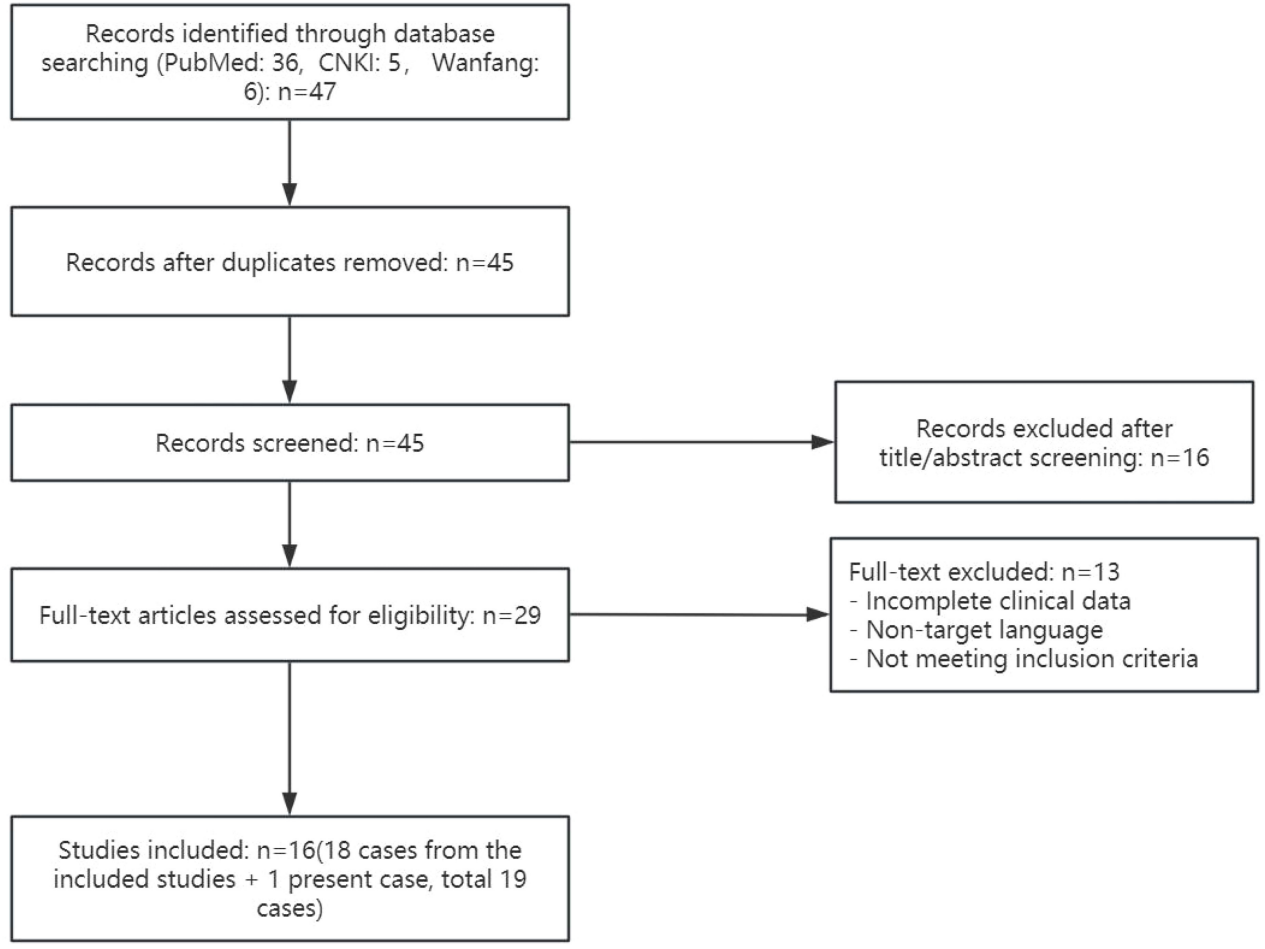
Flow diagram of literature screening for cola-induced hypokalemic paralysis.

We gathered data on the clinical characteristics of these cases from literature in online databases, including the CNKI database (Chinese), Wanfang Medical Database (Chinese), and PubMed database between January 1990 and December 2024. Search words include: hypokalemia, hypokalemia periodic paralysis, hypokalemia myopathy, cola, soft drink, carbonated drink”. Inclusion criteria: (1) case report or case series; (2) clearly record hypokalemia (blood potassium < 3.5 mmol/L); (3) report of drinking cola; (4) available clinical data. Exclusion criteria: (1) Incomplete clinical data; (2) Repeated reports; (3) Irrelevant to the research topic. The two authors independently screened the titles and abstracts, and retrieved a total of 16 articles (Figure 2). There were 19 cases of hypokalemia (including the present case), caused by drinking large amounts of cola, resulting in muscle weakness and hypokalemic paralysis (Supplementary Table 1). Of these, eight were women (including two pregnant cases), and 11 were men. The most common symptom was fatigue, and 10 cases involved limb paralysis/paresis. The causes were related to cola-induced hypokalemia, and most cases had severe hypokalemia. Nine hypokalemia cases (four men and five women) caused by drinking large amounts of cola were reported in Asia, while the remaining ones (seven men and four women) were in other continents. A comparative analysis of the clinical characteristics of cola-induced hypokalemia in Asian and non-Asian populations revealed no statistically significant differences in all clinical indicators (all *P* > 0.05, Supplementary Table 2). In the cases above, blood potassium levels returned to normal after potassium supplementation and cessation of cola intake.

**FIGURE 3:**
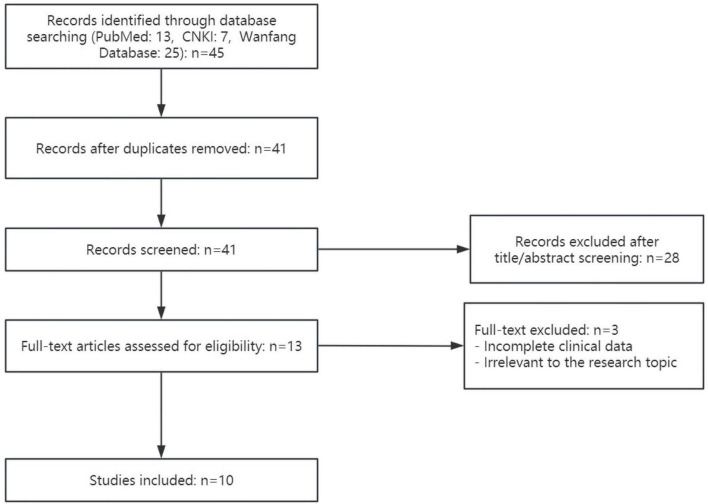
Flow diagram of literature screening for olanzapine-related hypokkalemic paralysis.

We gathered data on the clinical characteristics of these cases from literature in online databases, including the CNKI database (Chinese), Wanfang Medical Database (Chinese), and PubMed database between January 1990 and December 2024. Search words include: Olanzapine, Olanzapine Pamoate, hypokalemia, hypokalemic periodic paralysis, hypokalemic myopathy. Inclusion Criteria: (1) case report or case series; (2) clearly record hypokalemia (blood potassium < 3.5 mmol/L); (3) Subjects developed hypokalemic paralysis following olanzapine administration; (4) available clinical data. Exclusion criteria: (1) Incomplete clinical data; (2) Repeated reports; (3) Irrelevant to the research topic. The two authors independently screened the titles and abstracts, and retrieved a total of 10 articles (Figure 3). There were 12 cases of olanzapine-induced hypokalemia (three women and nine men) (Supplementary Table 3). Based on statistical analysis of data from patients with hypokalemic periodic paralysis caused by two inducements (Supplementary Table 4), the average blood potassium levels of patients with cola- and olanzapine-induced hypokalemia were 2.16 ± 0.57 mmol/L and 2.60 ± 0.49 mmol/L, respectively. There was a significant difference between patients with cola-induced hypokalemia versus those with olanzapine-induced hypokalemia (*P* = 0.033).

## Discussion

Hypokalemic periodic paralysis, which is characterized by recurrent, sudden skeletal muscle flaccid paralysis, accompanied by decreased blood potassium levels, is more common in men than women. The muscle weakness involves the limbs, and in severe patients complicated with respiratory muscle paralysis or arrhythmia, it can cause death. Potassium supplementation can rapidly relieve the symptoms of hypokalemic periodic paralysis without sequelae, but some patients have recurrent attacks. However, in this case, the patient was complicated with hypoglycemia at the time of consultation, had a history of type 2 diabetes and schizophrenia, and was being treated with olanzapine, which can also lead to low potassium. Follow-up examination revealed that the low potassium was caused by cola, and finally, the patient was successfully treated. Currently, the mechanism of hypokalemia related to long-term consumption of large amounts of cola is still unclear, and many viewpoints are mostly speculative. Cola may cause low blood potassium through the following mechanisms: (1) Insufficient potassium intake. Patients who drink a lot of cola, which has a low potassium content, tend to eat less, causing insufficient daily potassium intake. The World Health Organization recommends that adults get at least 3,510 mg of potassium per day. Four patients mentioned blood potassium, but their daily intake was far below the recommended amount. (2) Intracellular and extracellular redistribution of potassium in the body. The large amount of glucose in cola (2) can lead to hyperinsulinemia, which can cause extracellular potassium to enter the cell. Because cola is carbonated, excessive intake can lead to alkalosis and promote the transfer of extracellular potassium into cells. (3) High glucose levels can lead to osmotic diuresis and excessive urinary potassium excretion (3), reducing the concentration of extracellular potassium (i.e., blood potassium). Cola contains a high amount of fructose (13), and levels of unabsorbed fructose in the colon may cause osmotic diarrhea and aggravate potassium loss. According to the Chinese Dietary Guidelines (2022), it is recommended that daily added sugar intake should not exceed 50 g, and it’s best to keep it under 25 g. Drinking a lot of Coke has already exceeded this recommended amount. (4) Excessive caffeine intake. Cola contains caffeine, and excessive caffeine can induce respiratory alkalosis (14), cause increased catecholamine release, transfer extracellular potassium into the cell, induce renin release (15), and increase renal potassium excretion, leading to hypokalemia. Symptoms of caffeine toxicity may occur in adult at doses of as little as 500–600 mg/day. (5) Four patients consume more than 600 mg of caffeine per day.

In addition, our patient has not experienced weight loss and their BMI does not meet the criteria for malnutrition. However, one thing we haven’t checked is the patient’s muscle mass, which is also a criterion for diagnosing malnutrition. Given that the patient still feels weak after correcting potassium levels, they might have muscle-wasting malnutrition. Besides, the patient also has mild anemia (Hemoglobin level of 106 g/L) and low protein levels (albumin level of 31.8 g/L), which also suggest malnutrition. And malnutrition can also cause hypokalemia.

Most patients with hypokalemia have a mild to moderate reduction in serum potassium concentration. Patients with severe hypokalemia (serum potassium levels of <2.5 mmol/L) rarely present with arrhythmia and muscle necrosis, followed by rhabdomyolysis and ascending paralysis (16). Reduced potassium intake, extracellular-to-intracellular potassium transfer, and gastrointestinal or renal decompensation of serum potassium can cause hypokalemia. Several reports have shown that excessive cola consumption can lead to severe hypokalemia, accompanied by myopathy, rhabdomyolysis, acute kidney injury, and atrial fibrillation (3, 4, 9–11, 17). However, long-term consumption of high amounts of cola can cause severe hypokalemia, and there is a limited understanding of it. The first report that drinking a lot of cola causes severe hypokalemia (1) involved a 21-year-old pregnant woman who developed severe hypokalemia after drinking a lot of cola, with symptoms like fatigue and loss of appetite. Serum potassium levels returned to normal, and the symptoms were relieved after stopping cola intake. Several cases of hypokalemia caused by drinking large amounts of cola have since been reported (2–12, 17–20). These reports showed that large amounts of cola led to hypokalemia (with symptoms ranging from mild muscle weakness to paralysis), which improved after blood potassium levels returned to normal. A 2010 report (9) described a case of hypokalemia caused by long-term cola intake, secondary to rhabdomyolysis and acute kidney injury, which improved after hemodialysis. In a 2017 case report of a patient with rhabdomyolysis secondary to hypokalemia caused by long-term cola intake (11), the patient completely recovered after stopping cola intake and undergoing a month of potassium replacement therapy. The three cases reported in 2015 (12) showed that large amounts of cola can cause lower limb fatigue and abnormal renal tubular function, which could return to normal after ceasing cola intake (recovery time: 1 day to 3 months). Although the muscle strength of the patient reported in this article improved after potassium supplementation treatment, he did not fully recover until 2 months later, and his free lower limb movement was not restricted. Therefore, an unbalanced diet involving excessive consumption of cola not only causes lower limb fatigue but also has more serious consequences.

Currently, the cases of olanzapine-induced hypokalemia all come from China (Supplementary Table 3). Lower limb fatigue, including two cases of tachycardia (21, 22) and four cases of limb paralysis/soft paralysis, was the most common symptom (23–26). Reducing olanzapine dosage or stopping it returned blood potassium levels to normal and relieved myasthenic symptoms, with gradual recovery in all patients. Olanzapine, a thiophene benzodiazepine derivative, mainly acts on dopamine receptors, serotonin receptors, and other neurotransmitter receptors to regulate brain nerve activity. It is mainly used to treat schizophrenia and relieve hallucinations, confusion, and other symptoms. The mechanism by which olanzapine causes hypokalemia is unclear. It is thought to interfere with glucose metabolism, causing glycogen accumulation in cells and potassium ion entry into the cell through osmosis, thereby resulting in reduced levels of blood potassium (26). It may also interfere with the intestinal function, resulting in excessive potassium loss (27). The patient in this report did not have any episodes of hypokalemia during follow-up for half a year, and his hypokalemic paralysis was associated with an unbalanced diet involving excessive consumption of cola. We compared the clinical data of the cola group and the olanzapine group and found that the cola group had lower blood potassium levels. Since the data of these two groups came from the literature, the results still need more research to confirm. But before better studies appear in the future, this comparison gives us preliminary information. Schizophrenia patients using olanzapine who develop low blood potassium need to pay attention to whether their diet, such as cola, has an impact. We believe such cases are not rare, it’s just that people haven’t noticed. People with schizophrenia have a higher risk of metabolic problems and cognitive decline, and diet quality might be linked to metabolism and cognitive function. Compared to healthy people, their eating habits are usually not that great (28). Schizophrenia patients have poor nutritional patterns, used to consume more fats and sweet drinks frequently (29).

## Conclusion

There are few reports on hypokalemia related to long-term consumption of large amounts of cola, which has not attracted attention among clinicians. Therefore, it is easy to miss its diagnosis or misdiagnose it. Considering the popularity of cola globally, when doctors encounter patients with unexplained hypokalemia, they should understand the medical history of the patients and ask about their eating habits, especially cola intake. In addition to correcting hypokalemia, patients with hypokalemia caused by cola also need dietary suggestions and attention to prevent recurrence. It should be noted that hypokalemia associated with an unbalanced diet involving excessive consumption of cola causes other more serious complications, not just lower limb fatigue. Hypokalemia and other possible health problems can be prevented by educating the public and raising awareness of the possible health problems caused by an unbalanced diet. Clinicians should understand that lower limb fatigue may remain for a long time after correcting hypokalemia. When patients who drink a lot of cola while taking olanzapine develop hypokalemia, they should stop drinking cola to identify the cause and make corresponding adjustments to treatment with the drug. Moreover, although cola is the focus of our study, there have also been reports of hypokalemia caused by other beverages. A balanced diet is key to preventing and treating this disease.

## Data Availability

The original contributions presented in this study are included in the article/Supplementary material, further inquiries can be directed to the corresponding author.
