# Supplementary material for: A case and literature review of hypokalemic periodic paralysis associated with heavy cola consumption
Source: Front Nutr. 2026 Aug 4;13:1841484. doi: 10.3389/fnut.2026.1841484 (PMC13481175; doi:10.3389/fnut.2026.1841484)
Supplement: Supplementary file 1 [file Table_1.DOC]

**Supplement Table 1**. Summary of case reports on beverage-induced hypokalemic paralysis.

| **Case** | **Literature** | **Onset Age/sex** | **Country** | **symptoms** | **Daily Cola*** **intake**  **(L)** | | **Duration of drink intake** | **Daily Component Intake** | **Blood potassium**  **(mmol/L)** | **Urinary potassium**  **(mmol/L/d)** | **Renin**  **(ng/ml/h)** | **Aldosterone**  **(ng/dl)** | **Follow-up potassium levels after stopping/reducing beverage intake (mmol/L)** | **Muscle Strength** |
| --- | --- | --- | --- | --- | --- | --- | --- | --- | --- | --- | --- | --- | --- | --- |
| 1 | Matsunami et al  ^Error: Reference source not found^ | 21/F | Japan | - | 3 | 6 years | | - | 1.9 | - | - | - | N | - |
| 2 | Sharma and Guber^Error: Reference source not found^ | 58/M | America | loss of appetite and general weakness | 6–7 | - | | Caffeine: , 700–1100 mg/day | 2.4 | - | - | - | N | Ⅲ |
| 3 | Dubeyet al  ^[1]^ | 65/M | America | episodic paroxysmal lower limb paralysis | 3–4 | 2–3 years | | Contains carbonated water, sugar, colorant, phosphoric acid, caffeine | 1.9 | 26 | 2.1 | 5.6 | 3.9 | - |
| 4 | Lee et al  ^[3]^ | 52/M | Korea | progressive paralysis in both extremities | 4–5 | 1.5 months | | Caffeine: total ;1000 mg/day | 2.3 | 6.6 | N | N | 4.2 | Ⅳ |
| 5 | Packer^Error: Reference source not found^ | 52/M | America | generalized weakness and fatigue | 4 | several years | | fructose396 g/day ; K: 169 mg/day | 3 | 8.6 | 0.3 | 4.8 | 3.5 | Ⅳ |
| 6 | Appel CC,et al^[4]^ | 24/F | America | loss of appetite, myasthenia of limbs, and lower limb pain | 6–7 | 10 months | | Caffeine: 900 mg/day | 2 | 41.9 | 12.9 | 3.6 | N | - |
| 7 | Mudge D et  Al.^Error: Reference source not found^ | 44/M | Australia | tetraplegia | 4-10 | 3 years | | Sugar: 440-1100 g/day; Negligible potassiumCaffeine:388- 970 mg/L | 1.4 | 17.4 | N | N | N | Ⅱ |
| 8 | Rice JE et al Error: Reference source not found | 21/F | Australia | progressive proximal muscle weakness,  nausea, vomiting, diarrhea, and weight loss | 8 | 1–2 years | | Caffeine:, total ≥1000 mg/day | 1.8 | 9.0 | N | N | N | Ⅳ |
|  |  |  |  |  |  |  | |  |  |  |  |  |  |  |
| 9 | Ferrazzoli et al  ^[5]^ | 38/M | Italy | Progressive lower extremity proximal muscle weakness | 6 | 3 years | | Caffeine: 500–700 mg/day | 1.06 | 7.0 | - | - | N | Ⅱ |
| 10 | Takami et al  ^[9]^ | 43/F | Japan | myasthenia of limbs | 4–5 | 2 years | | Caffeine:500–600 mg/day | 1.8 | 22.3 | 0.2 | 0.019 | N | - |
| 11 | Yaguchi M[10] | 30/M | Japan | - | 1.5–2.5 | 3 months | | Caffeine: 180-300mg/L, | 2.3 | - | - | - | N | - |
| 12 | Su Xing et al^Error: Reference source not found^ | 26/F | China | Fatigue and muscle weakness of both lower limbs | 2 | 1 year | | - | 2.8 | 33.4 | N | N | 4.0 | Ⅲ |
| 13 | Su Xing et al^Error: Reference source not found^ | 36/F | China | Progressive fatigue | 2–2.5 | 3 years | | - | 2.6 | 39.5 | N | N | 3.9 | - |
| 14 | Su Xing et al^Error: Reference source not found^ | 18/M | China | hypodynamia | 2 | 2 years | | - | 3.0 | - | - | - | 4.0 | - |
| 15 | Franconieri et al ^[12]^ | 44/M | France | Bilateral myalgia of quadriceps femoris and shoulders | 3 | 2 years | | Sugar :315 g/day, Caffeine:285 mg/day, K<3mmol/day | 2.7 | 11.0 | - | - | N | Ⅲ |
| 16 | Baba-Aissa et al^[14]^ | 30s/M | Britain | central chest discomfort with  occasional palpitations, muscle spasms, and restless  ness | 3–3.5 | 5 years | | Sugar: 370 g/day,Trace amount of potassium | 2.8 | - | 0.67 | 6.9 | 3.5 | - |
| 17 | Madkhali et al^[15]^ | 62/F | America | progressive  muscular weakness associated with nausea, vomiting, and diarrhea | 3–5 | - | | HFCS: 420–520 g/day, Caffeine:380–470mg/day) | 1.6 | - | - | - | 4.0 | Ⅱ |
| 18 | Zakharova E^[16]^ | 36/F | Russia | Limb paralysis, vomiting, headache, polyuria, and arrhythmia | 2 | 20 years | | Sugar :210 g/day(usual) / 525g/day (onset); Caffeine:194 mg/day (usual) /485 mg/day(onset); | 1.4 | - | - | N | 4.5 | Ⅰ |
| 19 | Our case | 34/M | China | Hand shaking, dysthesia, with lower limb weakness | 4 | - | | **S**ugar :424 g/day, Caffeine：400mg/day  K:0mmol/day | 2.74 | 25.2 | N | N | N | Ⅳ |

N = value within normal range, - = not specified in the case report,K = potassium, M = male, F = female, HFCS = high-fructose corn syrup,.

In this article, 'cola' refers to carbonated soft drinks that contain caffeine and a large amount of sugar (glucose/fructose) and are called cola. Other soft drinks with similar ingredients that are not called cola are not included.

References

[1]Matsunami K, Imai A, Tamaya T. Hypokalemia in a pregnant woman with long-term heavy cola consumption. Int J Gynecol Obstet (1994) 44:283–4. doi: 10.1016/0020-7292(94)90182-1
[2]Sharma R, Guber HA. Cola-induced hypokalemia: a case report and review of the literature. Endocr Pract (2013) 19:e21–3. doi: 10.4158/EP12241.CR
[3]Dubey D, Sawhney A, Sharma A, Dubey D. Paroxysmal paralytic attacks secondary to excessive cola consumption. Clin Med Res (2014) 12:61–4. doi: 10.3121/cmr.2013.1167
[4]Lee HJ, Kim DW, Cho HS, Jung EY, Lee DW, Chang S-H, et al. Hypokalaemic paralysis induced by large amounts of cola consumption. Nephrol Dial Transplant (2007) 22:2729. doi: 10.1093/ndt/gfm355
[5]Packer CD. Chronic hypokalemia due to excessive cola consumption: a case report. Cases J. 2008 Jul 14;1(1):32. doi: 10.1186/1757-1626-1-32.
[6]Appel CC, Myles TD. Caffeine-induced hypokalemic paralysis in pregnancy. Obstet Gynecol (2001) 97:805–7. doi: 10.1097/00006250-200105000-00037
[7]Mudge DW, Johnson DW. Coca-Cola and kangaroos. Lancet (2004) 364:1190. doi: 10.1016/S0140-6736(04)17111-0
[8]Rice JE, Faunt JD. Excessive cola consumption as a cause of hypokalaemic myopathy. Intern Med J. 2001 Jul;31(5):317-8. doi: 10.1046/j.1445-5994.2001.00063.x. PMID: 11512607.
[9]Ferrazzoli D, Sabetta A, Palamara G, Caremani L, Capobianco M, Balbi P, et al. A cola-induced hypokalemic rhabdomyolysis with electromyographic evaluation: a case report. SAGE Open Med Case Rep (2017) 5. doi: 10.1177/2050313X17695717
[10]Takami M, Imuta N, Hara H, Abe Y, Oguro R, Shimaoka I, et al. Hypokalemic myopathy with long-term heavy cola consumption. J Jpn Soc Intern Med (2005) 94:132–4. doi: 10.2169/naika.94.132
[11]Yaguchi M, Yaguchi H. Hypokalemic myopathy due to excessive consumption of cola. Intern Med (2010) 49:1833. doi: 10.2169/internalmedicine.49.3995
[12]Xing S, Xiaohua L, Yafang W, Yang L. Hypokalemia caused by Coca-Cola: three cases report and literature review. Chin J Intern Med (2015) 54:882–4.
[13]Franconieri F, Oehler E, Grémain V. Un diagnostic servi sur un plateau tahitien [Myalgia in a 44 year-old man]. Rev Med Interne. 2024 Oct;45(10):662-664. French. doi: 10.1016/j.revmed.2024.05.021. Epub 2024 Jul 17. PMID: 39025688.
[14]Baba-Aissa S, Cooke L, Alner V, Ahmed MH. Hypokalaemia associated with excessive cola-flavoured drinks consumption. BMJ Case Rep. 2024 Apr 22;17(4):e258873. doi: 10.1136/bcr-2023-258873. PMID: 38649244; PMCID: PMC11043727.
[15]Madkhali MA. Reversible Takotsubo Syndrome Complicated With Acute Thromboembolic Stroke Co-Occurring With Cola-Induced Severe Hypokalemia. Cureus. 2021 Jul 18;13(7):e16470. doi: 10.7759/cureus.16470.
[16]Zakharova E: Severe hypokalemia due to excessive coca-cola consumption, presenting with tetraparesis, rhabdomyolysis, atrial fibrillation and hypokalemic nephropathy - case report. J Urol Nephrol Open Access. 2016, 2:1-4. 10.15226/2473-6430/2/2/00114
