# Supplementary material for: A case and literature review of hypokalemic periodic paralysis associated with heavy cola consumption
Source: Front Nutr. 2026 Aug 4;13:1841484. doi: 10.3389/fnut.2026.1841484 (PMC13481175; doi:10.3389/fnut.2026.1841484)
Supplement: Supplementary file 2 [file Table_2.DOC]

**Supplement Table 2**. Clinical characteristics of hypokalemia caused by cola in Asia and non-Asian countries.

|  | Asia | non-Asia | P |
| --- | --- | --- | --- |
| Male/Female(example) | 4/4 | 7/4 | 0.578 |
| Age at onset(years) | 32.50±11.31 | 44.40±15.10 | 0.083 |
| Blood potassium（mmol/L) | 2.43±0.43 | 1.96±0.59 | 0.075 |
| Mild muscle weakness/Severe muscle weakness(example) | 8/0 | 7/4 | 0.103 |
| Follow-up potassium levels after stopping/reducing beverage/olanzapine intake (mmol/L) | N | N | -- |

N = value within normal range, - = not specified in the case report. K = potassium, M = male, F = female.
