# Supplementary material for: A case and literature review of hypokalemic periodic paralysis associated with heavy cola consumption
Source: Front Nutr. 2026 Aug 4;13:1841484. doi: 10.3389/fnut.2026.1841484 (PMC13481175; doi:10.3389/fnut.2026.1841484)
Supplement: Supplementary file 3 [file Table_3.DOC]

| **Supplement table 3** Summary of Case Reports of Olanzapine induced hypokalemic paralysis | | | | | | | | | |
| --- | --- | --- | --- | --- | --- | --- | --- | --- | --- |
| case | Literature | Onset Age/sex | Blood potassium levels before starting/increasing intake of olanzapine (mmol/L) | Olanzapine Intake (mg/d) | Duration of Olanzapine intake | Blood assium level after olanzapine intake (mmol/L) | Muscle Strength | | Follow up potassium levels after stopping/reducing Olanzapine intake (mmol/L) |
|  |  |  |  |  |  |  | upper limb | lower limb |  |
| 1 | Gai Qiong , et al^[1]^ | 17/M | - | 15 | 12 days | 2.21 | Ⅴ^－^ | Ⅱ | N |
| 2 | Tao Hong,et al.^[2]^ | 85/F | - | 5 | Decades | 2.64 | Ⅳ | Ⅲ | 3.77 |
| 3 | Fang Liping^[3]^ | 43/M | 4.4 | 10 | 20 days | 3.1 | - | Ⅲ | 4.0 |
| 4 | Zou Qingmei^[4]^ | 31/M | - | - | - | 1.95 | Ⅲ | Ⅱ | 5.24 |
| 5 | Zhang Deliang,Meng Qingling^[6]^ | 36/M | 3.7 | 10 | 7 weeks | 1.8 | - | Ⅱ | N |
| 6 | Lin Wensheng^Error: Reference source not found^ | 54/M | - | 5 | 5 days | 3.13 | - | - | N |
| 7 | Lin Wensheng^Error: Reference source not found^ | 55/M | - | 7.5 | 6 days | 2.1 | - | - | N |
| 8 | Li Hualin^[5]^ | 24/M | 3.6 | 5 | 6 days | 2.5 | - | Ⅳ | 4.74 |
| 9 | Li Hualin^[5]^ | 28/M | - | 5 | 6 days | 2.93 | - | Ⅳ | 3.8 |
| 10 | Huang G et al^[9]^ | 75/F | - | 5 | 3 months | 3.11 | - | - | 4.25 |
| 11 | Guo Li ,et al^[8]^ | 47/M | - | 5 | 4 months | 2.7 | - | Ⅱ | N |
| 12 | Fu Qiuyu,et al.^[10]^ | 68/F | - | 5 | 4 months | 3.06 | - | - | 4.29 |

N = value within normal range;-= not specified in the case report. K=Potassium; M=male, F=female

**References**

[1]Qiong G,Tiefeng W, Chao Q, Jiongjiong X. Olanzapine-induced severe hypokalemia in one adolescent[J]. Chinese Journal of Primary Medicine and Pharmacy, 2021, 28(11): 1723-1725.doi:10.3760/cma.j.issn.1008-6706.2021.11.027.
[2]Hong T,Guowen C,Aiming S, Junjie B. Pharmaceutical care of olanzapine-induced hypokalemia and atypical malignant syndrome[J]. Herald of Medicine, 2015, 34(04): 553-555.doi:10.3870/yydb.2015.04.039.
[3]Liping F. Hypokalemic paralysis induced by olanzapine in 1 case[J]. Journal of Neuroscience and Mental Health, 2003, 3(4): 277-277.doi:10.3969/j.issn.1009-6574.2003.04.040.
[4]Qingmei Z. Hypokalemic paralysis induced by olanzapine in 1 case[J]. Evaluation and Analysis of Drug-Use in Hospitals of China, 2013, 13(12): 1152.doi:10.14009/j.issn.1672-2124.2013.12.011
[5]Hualin L. Report of two cases of hypokalemia caused by olanzapine[J]. Chinese Journal of Drug Abuse Prevention and Treatment, 2017, 23(03): 168-169.doi:10.15900/j.cnki.zylf1995.2017.03.010.
[6]Deliang Z,Qingling M. One case of severe hypokalemia caused by olanzapine[J]. CHINESE JOURNAL OF CLINICAL PHARMACY, 2000(05): 315. doi:10.3969/j.issn.1007-4406.2000.05.027.
[7]Lin Wensheng L. Olanzapine caused hypokalemia in 2 cases[J]. Chinese Journal of Pharmacoepidemiology, 2006(05): 314.doi:10.3969/j.issn.1005-0698.2006.05.030.
[8]Li G,Huaihai W ,JYuting J, Qingrong T.One case of hypokalemic paralysis caused by olanzapine[J]. Journal of Clinical Psychiatry, 2010, 20(03): 148.
[9]Huang G, Fu Q, Xu J. Potential torsades de pointes triggered by hypokalemia related to olanzapine in a patient with implantable cardioverter-defibrillator. J Clin Psychopharmacol. 2014,34(5):651-2. doi: 10.1097/JCP.0000000000000189.
[10]Qiuyu F, Junbo X,Yongmei H, et al. Olanzapine-induced hypokalemia induced torsades de pointes ventricular tachycardia : a case report[J]. Chinese Journal of Practical Internal Medicine, 2013, 33(05): 411-412+414.
