# Supplementary material for: A case and literature review of hypokalemic periodic paralysis associated with heavy cola consumption
Source: Front Nutr. 2026 Aug 4;13:1841484. doi: 10.3389/fnut.2026.1841484 (PMC13481175; doi:10.3389/fnut.2026.1841484)
Supplement: Supplementary file 4 [file Table_4.DOC]

**Supplement Table 4.** Clinical characteristics of cola-induced and olanzapine-induced hypokalemia.

|  | Cola-induced hypokalemia | Olanzapine-induced hypokalemia | P |
| --- | --- | --- | --- |
| Male/Female(example) | 11/8 | 9/3 | 0.254 |
| Age at onset(years) | 39±15 | 47±21 | 0.241 |
| Blood potassium (mmol/L) | 2.16±0.57 | 2.60±0.49 | 0.033 |
| Mild muscle weakness/Severe muscle weakness (example) | 15/4 | 8/4 | 0.676 |
| Follow-up potassium levels after stopping/reducing beverage/olanzapine intake (mmol/L) | N | N | -- |

N = value within normal range, - = not specified in the case report, K = potassium, M = male, F = female.
